# Co-regulation of Thermosensor Pathogenic Factors by C-di-GMP-Related Two-Component Systems and a cAMP Receptor-like Protein (Clp) in *Stenotrophomonas maltophilia*

**DOI:** 10.3390/foods13081201

**Published:** 2024-04-15

**Authors:** Jieqiong Ding, Minghong Liao, Qingling Wang

**Affiliations:** Shaanxi Natural Carbohydrate Resource Engineering Research Center, College of Food Science and Technology, Northwest University, Xi’an 710069, China; 202323969@stumail.nwu.edu.cn (J.D.); 202233799@stumail.nwu.edu.cn (M.L.)

**Keywords:** *Stenotrophomonas maltophilia*, two-component system, c-di-GMP metabolism, biofilm, Clp-mediated

## Abstract

*Stenotrophomonas maltophilia* is a major threat to the food industry and human health owing to its strong protease production and biofilm formation abilities. However, information regarding regulatory factors or potential mechanisms is limited. Herein, we observed that temperature differentially regulates biofilm formation and protease production, and a cAMP receptor-like protein (Clp) negatively regulates thermosensor biofilm formation, in contrast to protease synthesis. Among four c-di-GMP-related two-component systems (TCSs), promoter fusion analysis revealed that *clp* transcription levels were predominantly controlled by LotS/LotR, partially controlled by both RpfC/RpfG and a novel TCS Sm0738/Sm0737, with no obvious effect caused by Sm1912/Sm1911. Biofilm formation in Δ*clp* and ΔTCSs strains suggested that LotS/LotR controlled biofilm formation in a Clp-mediated manner, whereas both RpfC/RpfG and Sm0738/Sm0737 may occur in a distinct pathway. Furthermore, enzymatic activity analysis combined with c-di-GMP level indicated that the enzymatic activity of c-di-GMP-related metabolism proteins may not be a vital contributor to changes in c-di-GMP level, thus influencing physiological functions. Our findings elucidate that the regulatory pathway of c-di-GMP-related TCSs and Clp in controlling spoilage or the formation of potentially pathogenic factors in *Stenotrophomonas* expand the understanding of c-di-GMP metabolism and provide clues to control risk factors of *S. maltophilia* in food safety.

## 1. Introduction

*Stenotrophomonas maltophilia*, previously known as *Pseudomonas maltophilia* or *Xanthomonas maltophilia*, is an aerobic, gram-negative, non-fermentative bacillus that is ubiquitous in various aqueous, clinical, and ecological environments, including plant roots, food, soil, and plant stems [1,2,3,4,5]. The organism has long been considered a major problem in the hospital setting and can cause cystic fibrosis of the lungs and other diseases, including infections of the skin and soft tissue, bloodstream, and urinary tract [6,7,8]. Moreover, *S. maltophilia* has emerged as a global opportunistic human pathogen and has been characterized by multi-resistance to many antibiotics [3,9,10,11]. In the food industry, *S. maltophilia* occurs in diseased fish and various foods, such as raw milk [12], spoiled vegetables [13], frozen dumplings, marine food products [14], and even drinking water [15]. The presence of *S. maltophilia* in food products causes spoilage and seriously threatens human health. 

Recent evidence indicates that protease activity and biofilm-forming ability are vital pathogenic factors and potential spoilage determinants of *S. maltophilia* [10,16,17]. In a clinical study, extracellular proteases of *S. maltophilia* were proven to degrade human proteins and contribute to protease-mediated innate immune dysfunction in cystic fibrosis, tissue damage, and inflammation in patients’ lungs [17,18,19]. In food preservation, *S. maltophilia* produces a high level of proteases to decompose proteins in food and produce biogenic amines, forming an unpleasant smell and eventually resulting in food spoilage [20]. Biofilm-induced resistance developed in bacterial pathogens impacts human health seriously worldwide [21]. The treatment of *S. maltophilia* infection is very difficult and is due to its powerful ability to form biofilms, which leads to increased resistance to antimicrobial agents [10,22,23]. The major components of the biofilm matrix in most bacterial species include exopolysaccharide (EPS), lipopolysaccharide (LPS), extracellular DNA (eDNA), adhesions, and pili [24,25,26,27,28]. Bacterial swimming motility, EPS production, and LPS synthesis are usually related to biofilm formation and are critical aspects of bacterial spoilage and pathogenesis [29,30]. In the food industry, exploring the regulatory factors associated with biofilm formation and spoilage in *S. maltophilia* is of great significance for extending the shelf life of food.

Generally, the biofilm formation ability and food spoilage potential of *S. maltophilia* may be affected by external environments. The level of second messenger cyclic diguanylate (c-di-GMP) plays a critical role in bacterial adaptation to environmental changes, including cell swimming ability, biofilm formation, cell differentiation, and virulence [31,32]. The c-di-GMP level can be determined by two critical metabolic enzymes: phosphodiesterase containing an EAL or HD-GYP domain responsible for c-di-GMP degradation and diguanylate cyclase with a GGDEF domain for its synthesis [9,33,34,35]. Two-component systems (TCSs) link environmental signals to cellular responses and further control gene expression. According to NCBI sequence analysis, four TCSs involved in c-di-GMP metabolism were present in the genomes of *S. maltophilia*, and their regulatory functions during biofilm formation and other cell processes are still limited. In our previous study, we found that a cAMP receptor-like protein (Clp) controlled low temperature-dependent protease synthesis, while both TCSs (LotS/LotR and RpfC/RpfG) in *S. maltophilia* play critical roles in positively mediating protease production [36,37].

In this study, we analyzed *S. maltophilia* biofilms grown at different temperatures. The effect of Clp and four c-di-GMP related TCSs on biofilm formation and protease production at different temperatures was determined, and *clp* promoter fusion analysis in TCSs mutant strains was also investigated for further exploring the correlation regulation of c-di-GMP-related TCSs and Clp. In addition, the enzymatic functionality of the novel regulator Sm0737 was characterized in vitro, combining with the effect of Sm0737 on the c-di-GMP level overall to expand our understanding of c-di-GMP metabolism and function. This work not only elucidates the regulatory correlation of c-di-GMP-related TCSs and Clp on pathogenic and spoilage-related factors but also provides strategies to control these risk factors of *S. maltophilia* in clinical practice and the food industry. 

## 2. Materials and Methods

### 2.1. Bacterial Strains and Growth Conditions

The *Escherichia coli* JM109 strain was used in this study for gene cloning and the S17-1λpir for strain conjugation. The *S. maltophilia* strain FF11 and various gene deletion strains, including ∆*lotS*, ∆*lotR*, ∆*rpfC*, ∆*rpfG*, ∆*Sm0737*, and ∆*Sm1912*, were grown at the required temperatures (15, 25, or 37 °C). A Luria–Bertani (LB) medium was used to culture all strains. The concentrations of ampicillin, kanamycin, and tetracycline for *E. coli* were 100, 50, and 15 µg/mL, respectively. For *S. maltophilia* FF11, the concentrations of tetracycline, chloramphenicol, and ampicillin were 125, 150, and 50 µg/mL, respectively.

### 2.2. Plasmids and Strain Construction

The strains and plasmids used in this study are listed in Table 1. Deletion mutants of Sm1912 and Sm0737 were constructed using the pEX18Tc vector based on homologous recombination, as previously described [38]. The FF11 genome was used to amplify the upstream and downstream gene fragments of *Sm1912* or *Sm0737*, and the chloramphenicol resistance gene was amplified from the plysS plasmid. The overlapping PCR products of these three fragments were digested with *Eco*RI and *Hind*III and then inserted into the pEX18Tc plasmid, producing the plasmids pEX18Tc-*Sm1912* and pEX18Tc-*Sm0737*. The primers used are listed in Table 2. These recombinant vectors were transformed into the strain FF11 via conjugation. Individual colonies selected on LB solid plates were further validated via PCR and sequenced according to a previously described method [36,38,39]. To construct complementation strains, gene fragments containing the coding regions of *Sm1912* and *Sm0737* were amplified via PCR and cloned into the expression vector pLAFR3. These recombinant vectors were transferred into the respective mutant strains via conjugation.

### 2.3. Biofilm Assay and Quantification

Crystal violet staining was used to quantify the *S. maltophilia* biofilm mass, as described previously [40,41]. The wild-type (WT) FF11 strain and mutant strains were cultured overnight at 25 °C in a culture medium containing the appropriate antibiotics, and the cultures of WT and mutant strains were inoculated into each well of 96-well polystyrene plates containing LB medium and incubated at different temperatures (15, 25, or 37 °C) without shaking. The strain cultures were stained with a 0.1% crystal violet solution for 20 min and then washed rigorously with water, followed by solubilization of the crystal violet stain using absolute ethanol. The biofilm mass was determined by measuring the absorbance at 590 nm (Thermo Varioslan Flash 3001). The biofilm-forming ability of the strains on test tube walls was also investigated. Briefly, WT and mutant strains were grown at 25 °C in LB medium for 48 h in a test tube with shaking at 180 rpm, and biofilm formation was observed.

### 2.4. Swimming Motility and EPS Production

The swimming motility of *S. maltophilia* and its derivative strains was measured as previously reported [42], with modifications. The assayed strains were grown overnight at 25 °C in LB medium supplemented with the appropriate antibiotics and diluted to an OD_600_ of 0.3. The diluted cultures (0.5 μL) were then used to inoculate LB medium plates containing 0.15% agar. The strains were incubated at 25 °C for 48 h, and the diameters of the swimming zones were measured.

To determine the production of EPS, the strains were cultured at 25 °C in LB medium for 48 h (OD_600_ = 2.2). After centrifugation at 15,000× *g* for 30 min, the supernatants were collected, followed by adding two volumes of absolute ethanol. The culture supernatants/absolute ethanol mixtures were kept at −20 °C for 1 h. The EPS molecules were then precipitated and dried overnight in a 55 °C oven, and then the dry weight was measured.

For the plate assay, the strains were cultured in LB medium until the OD_600_ reached 0.8. One microliter of each culture was spotted onto LB agar plates without antibiotics and incubated at 25 °C for 48 h. Afterwards, photographs of the colonies were taken. The experiments were independently repeated three times.

### 2.5. Analysis of Promoter Activity

*S. maltophilia* FF11 and mutant strains carrying the fusion plasmid pL6*rmlA*-gusA were cultured overnight at 25 °C in LB medium supplemented with the appropriate antibiotics. Cultures of these strains were then inoculated quantitatively into 30 mL of LB medium and cultured at 25 °C for 48 h. Bacterial cells were sampled and assayed for β-glucuronidase (gusA) activity using a method described in our previous study [36].

### 2.6. Bioinformatics Analysis

The predicted Sm0737 and Sm1912 proteins were characterized using BLAST searches in GenBank, together with the Conserved Domain Database, PFAM, and SMART programs. Multiple protein alignments were generated using ClustalW 2.0.10 software. Sequence comparison of the EAL domains of proteins with PDE enzymatic activity, including RocR, BifA, TdEAL, and YahA.

### 2.7. Enzymatic Activity Assays

The *Sm0737* gene fragment was cloned into the pLAFR3 plasmid, resulting in the recombinant plasmid pLAFR3-*Sm0737*, which was then transferred to a WT strain via conjugation. To express the Sm0737 protein, FF11 strains carrying the pLAFR3-*Sm0737* plasmid and expressing a C-terminal His6-tag were cultured at 25 °C for 48 h. Strain FF11 carrying the empty vector pLAFR3 was used as the control. After centrifugation at 7000× *g* for 10 min, the bacterial cells were collected and resuspended, followed by ultrasound treatment. The supernatant was loaded onto a HisTrap Excel column (GE, America) and eluted with different concentration gradients of buffer B (20 mM Tris-HCl, pH 7.5, 500 mM NaCl, and 500 mM imidazole). The eluate containing the Sm0737 protein was concentrated and further analyzed by SDS-PAGE.

The enzymatic assay for the Sm0737 protein was carried out as previously described [36]. Briefly, Sm0737 protein was incubated with the phosphodiesterase substrate bis(p-nitrophenyl) phosphate (5 mM) in an assay buffer (50 mM Tris-Cl, 100 mM NaCl, 10 mM MgCl_2_, pH 7.5) at 37 °C for 60 min. Enzyme activity was determined based on the hydrolysis of bis(p-nitrophenyl) phosphate to the yellow-colored compound p-nitrophenyl. The absorbance at 410 nm was measured using a spectrophotometer (Thermo Varioslan Flash 3001).

### 2.8. Detection of Intracellular c-di-GMP Levels

Liquid chromatography-tandem mass spectrometry (LC-2030, Shimadzu, Kyoto, Japan) was used to investigate the intracellular levels of c-di-GMP in strain FF11 and its various mutant strains, as described previously [36].

### 2.9. Statistical Analysis

All experiments were performed at least in triplicate, and the data were expressed as the mean ± standard deviation (SD). *p*-values less than 0.05, 0.01, and 0.001 were considered statistically significant and marked with “*”, “**”, and ***, respectively, *p*-values over 0.05 were considered non-significant and marked with “ns”. Statistical analysis of the data was performed through a one-way Analysis of Variance (ANOVA) test using SPSS Software (v15.0, SPSS Inc., Chicago, IL, USA). GraphPad Prism 6 software was used to generate the graphs.

## 3. Results

### 3.1. Temperature Differentially Regulates Biofilm Formation and Protease Production in S. maltophilia

**Figure 1:**
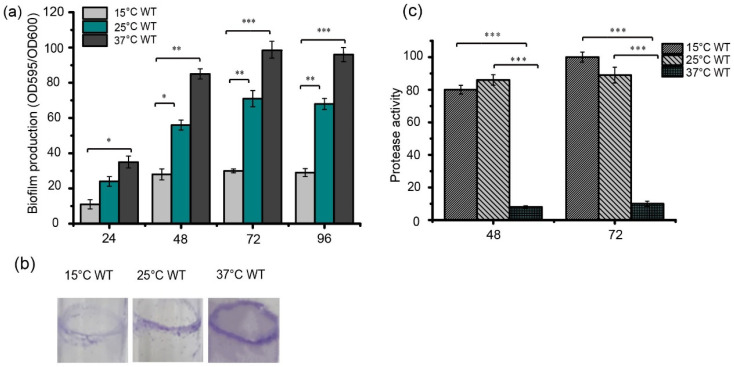
Temperature-dependent biofilm formation in *S. maltophilia* FF11. (**a**) Quantification of biofilm formation using OD_590_ measurements. Error bars represent the standard deviations (*n* = 3). Bacterial cells from cultures grown overnight in LB medium were collected and adjusted to an OD_600_ of 0.3. Equal volumes of bacterial cultures (10 μL) were inoculated into 96-well plates containing 190 μL of fresh LB medium. The plates were then incubated at 15, 25, and 37 °C at different times (24, 48, 72, and 96 h), and the biofilm quantities were calculated using the traditional crystal violet staining method. (**b**) Biofilm formation was observed on the test tube wall. The FF11 strain was cultured at 25 °C for 48 h in a test tube with shaking at 180 rpm. Biofilm formation was analyzed using crystal violet staining. (**c**) Protease activity of *S. maltophilia* FF11 cultured in a 180 rpm shaker at 15, 25, and 37 °C at different times (48 h and 72 h). Asterisks *, **, or *** represent a statistically significant difference between control and experi-ments at *p* < 0.05, *p* < 0.01, or *p* < 0.001, respectively.

Higher levels of biofilm formation were observed when the bacteria were grown at 25 °C and 37 °C compared to 15 °C, when these strains were inoculated into 96-well plates based on the crystal violet staining method (Figure 1a). In addition, the ability of strain FF11 to form biofilms was investigated by growing them in LB for 48 h at 25 °C with shaking at 180 rpm. The most obvious biofilms (purple stripes) were observed at 37 °C, then at 25 °C, and the lightest purple stripes were observed at 15 °C (Figure 1b). Protease activity was detected in the strains grown in LB medium at the required temperatures for 48 or 72 h (Figure 1c). Strain FF11 cultured at 15 °C or 25 °C had higher protease activity than strains cultured at 37 °C. These results indicate that temperature differentially regulates biofilm formation and protease synthesis in *S. maltophilia* FF11.

### 3.2. Clp Negatively Regulates Thermosensor Biofilm Formation in S. maltophilia

**Figure 2:**
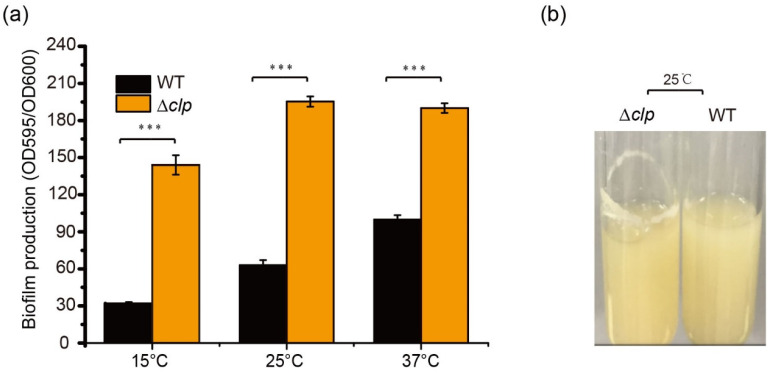
Clp negatively regulates biofilm formation in *S. maltophilia* FF11. (**a**) Quantification of biofilm formation in the wild-type (WT) and Δ*clp* mutant strains cultured at different temperatures (15, 25, and 37 °C) for 48 h. (**b**) Biofilm formation on the test tube wall. The WT and Δ*clp* strains were cultured at 25 °C for 48 h in a test tube under shaking at 180 rpm, and biofilm formation was observed. Asterisks *** represent a statistically significant difference between control and experiments at *p* < 0.001.

To determine the function of Clp on biofilm formation in *S. maltophilia*, biofilm amounts were quantified using crystal violet staining in WT and Δ*clp* strains at different temperatures in LB after 48 h of culturation. There was a significant difference in biofilm amounts between Δ*clp* and WT strains; the biofilm of Δ*clp* strains increased significantly, demonstrating increases of 4.1-fold at 15 °C, 3.1-fold at 25 °C, and 1.7-fold at 37 °C (Figure 2a). In addition, we observed more obvious biofilm stripes for Δ*clp* strains compared with the WT strains when they were cultured in LB at 25 °C for 48 h (Figure 2b). These results indicated that Clp negatively regulated biofilm formation in *S. maltophilia*, with a contrast effect on protease synthesis.

### 3.3. LotS/LotR Negatively Mediates Thermosensor Biofilm Formation in S. maltophilia

**Figure 3:**
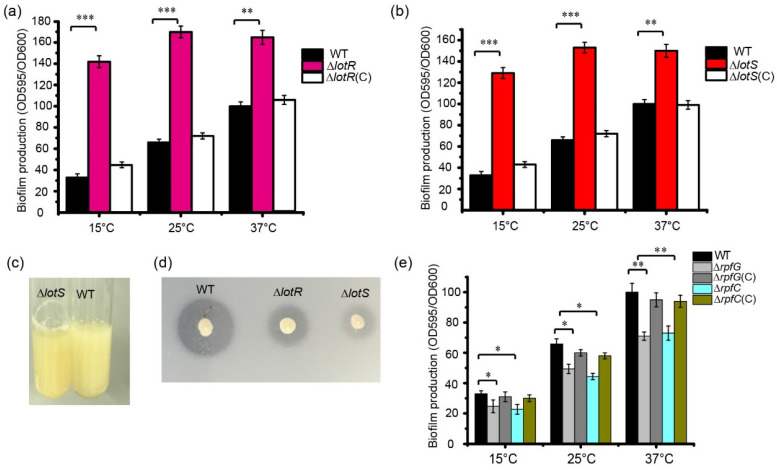
LotS/LotR and RpfC/RpfG-controlled thermosensor biofilm formation. Quantification of biofilm formation in the wild-type (WT), Δ*lotR*, Δ*lotR*(*C*) (**a**), and WT, Δ*lotS*, and Δ*lotS*(*C*) mutant strains (**b**) cultured at different temperatures (15, 25, and 37 °C) for 48 h. (**c**) Biofilm formation on the test tube wall. The WT and Δ*lotR* strains were cultured at 25 °C for 48 h in a test tube under shaking at 180 rpm, and biofilm formation was observed. (**d**) The protease activity of the WT and Δ*lotR* or Δ*lotS* strains on skim-milk agar plates. (**e**) RpfC/RpfG positively mediated thermosensor biofilm formation in the FF11 strain. The graph shows the quantification of biofilm formation for the wild-type (WT), Δ*rpfG*, Δ*rpfG*(C), Δ*rpfC*, and Δ*rpfG*(C) strains cultured at 15, 25, and 37 °C for 48 h. Asterisks *, **, or *** represent a statistically significant difference between control and experiments at *p* < 0.05, *p* < 0.01, or *p* < 0.001, respectively.

To evaluate the function of the LotS and LotR proteins in biofilm formation by *S. maltophilia*, the biofilm production of the WT and mutant strains was quantified using crystal violet staining. Deleting the *lotS* or *lotR* genes significantly increased biofilm production in *S. maltophilia* FF11. After 48 h of cultivation at different temperatures, compared to the WT strain, biofilm formation increased approximately 3.5–3.9-fold in Δ*lotR* or Δ*lotS* mutant strains cultured at 15 °C, approximately 2.1–2.4-fold higher for mutant strains cultured at 25 °C, and approximately 1.5–1.6-fold higher in mutant strains cultured at 37 °C (Figure 3a,b). We observed more obvious biofilm stripes for *lotR* mutant strains compared with WT strains when they were cultured in LB at 25 °C for 48 h (Figure 3c). Smaller clearing zones around the bacteria on the skim-milk agar plates were clearly observed in the *lotS* or *lotR* mutant strains compared to the WT strains (Figure 3d), verifying that LotS/LotR positively regulates protease activity in *S. maltophilia* FF11. These results indicated that LotS/LotR negatively regulate thermosensor biofilm formation, with a similar effect of Clp in *S. maltophilia* FF11.

### 3.4. RpfC/RpfG Positively Regulate Thermosensor Biofilm Formation in S. maltophilia

Compared with the LotS/LotR signal system, which significantly increased biofilm formation, the typical TCS, RpfC/RpfG, reduced the amount of biofilm (Figure 3e). Biofilm production was reduced by approximately 20% in *rpfC* or *rpfG* mutants compared with the WT strain when cultured at 37 °C. Approximately, a 13% decrease in biofilm formation was observed in both mutant strains when cultured in LB at 15 °C or 25 °C (Figure 3e).

### 3.5. LotS/LotR Contributes More to Physiological Functions Than RpfC/RpfG

**Figure 4:**
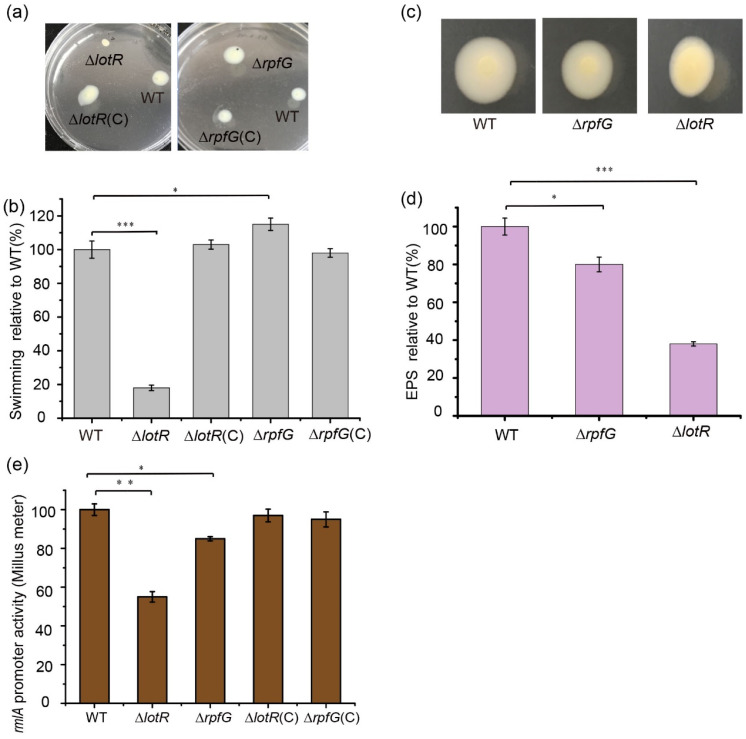
Effect of RpfC/RpfG and LotS/LotR on bacterial swimming and exopolysaccharide (EPS) and lipopolysaccharide (LPS) synthesis. Bacterial swimming motility was investigated in the wild-type (WT), Δ*rpfG*, Δ*rpfG*(C), Δ*rpfC*, and Δ*rpfC*(C) strains cultured at 25 °C in Luria–Bertani (LB) plates containing 0.15% agar. The diameters of the swimming zones were observed (**a**) and measured (**b**). (**c**) EPS production was observed based on the colony morphology. The WT, Δ*rpfG*, Δ*rpfG*(C), Δ*lotR*, and Δ*lotR*(*C*) strains were inoculated on LB plates containing 0.15% agar and cultured at 25 °C for 48 h. (**d**) Quantification of EPS production for the WT and mutant strains. (**e**) The effect of the regulators LotR and RpfG on *rmlA* expression. Strains carrying the fusion plasmid were cultured in LB medium with the appropriate antibiotics for 48 h at 25 °C, and the supernatant was used to detect β-glucuronidase activity. Asterisks *, **, or *** represent a statistically significant difference between control and experiments at *p* < 0.05, *p* < 0.01, or *p* < 0.001, respectively.

To better understand the regulatory mechanism of two distinct TCSs, RpfC/RpfG and LotS/LotR, on biofilm production, the effects of the response regulators RpfG and LotR on EPS production, bacterial swimming, and LPS synthesis were investigated. Deletion of the *lotR* gene positively controlled the ability of bacteria to swim, and a dramatic reduction in the bacterial expansion zone was observed (Figure 4a,b). A slight increase in the bacterial expansion zone showed that RpfG negatively mediated bacterial swimming motility (Figure 4a,b). Colonies of the WT FF11 strain displayed a smooth and glossy surface owing to the synthesis of large amounts of EPS. Comparatively, the Δ*lotR* mutant strain showed a drier and flatter colony surface compared with that of the Δ*rpfG* mutant (Figure 4c). Quantification of EPS synthesis exhibited that the deletion of the *lotR* gene reduced EPS amounts to approximately 64% of the WT strain. Approximately 20% less EPS synthesis was observed in the Δ*rpfG* mutant strain compared to the WT strain (Figure 4d), which was consistent with the colony morphology. Both the amounts of secreted EPS of the full-length complemented Δ*rpfG* (pLAFR3-*rpfG*) or Δ*lotR* (pLAFR3-*lotR*) strains were fully reverted to WT levels, as expected. These results suggest that the LotS/LotR regulatory system contributes more to various physiological functions in *S. maltophilia* compared to RpfC/RpfG.

To determine whether LotS/LotR and RpfC/RpfG could influence the expression of the *rmlA* gene, the promoter activity of *rmlA* was investigated in the respective mutants. The results revealed that β-glucuronidase activity was significantly reduced in Δ*lotR* mutants (45%), and only a slight reduction was observed in Δ*rpfG* mutants; complementing these proteins restored their expression to WT levels (Figure 4e). These results indicate that LotS/LotR may play a more important role in LPS synthesis than RpfC/RpfG.

### 3.6. S. maltophilia Encodes Two Additional TCSs Related to c-di-GMP Metabolism

Based on a database of prokaryotic TCSs, the genome of *S. maltophilia* contains approximately four putative TCSs containing GGDEF, EAL, and HD-GYP domains, which may be related to c-di-GMP metabolism. In addition to the LotS/LotR identified by our group and the classic quorum sensing system RpfC/RpfG (Sm1829/Sm1828) widely studied in many bacteria, two additional TCSs, Sm1911/Sm1912 and Sm0738/Sm0737, are also present in *S. maltophilia*. Although Sm1912 shares an 83% sequence similarity with the RavR protein of the plant pathogen *Xanthomonas* [43], *S. maltophilia* is not a plant pathogen, and these virulence-related regulators may show subtle functional differentiation in signal detection between *Stenotrophomonas* and *Xanthomonas*.

**Figure 5:**
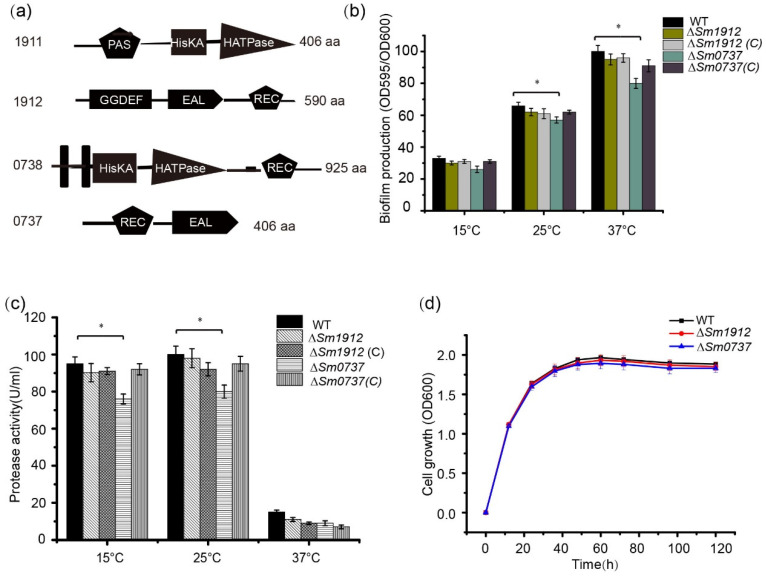
The effect of Sm0737 and Sm1912 on protease production and biofilm formation. (**a**) Schematic representation of the predicted domain structures of Sm0737 and Sm1912. Quantification of biofilm formation (**b**) and protease production (**c**) in the wild-type (WT), Δ*Sm0737*, Δ*Sm0737*(*C*), Δ*Sm1912*, and Δ*Sm1912*(*C*) strains. (**d**) Growth curves of the WT, Δ*Sm0737*, and Δ*Sm1912* strains and their complementary strains at 15, 25, and 37 °C. Asterisks * represent a statistically significant difference between control and experiments at *p* < 0.05.

Another TCS was composed of the signal sensor protein Sm0738, which contains a C-terminal catalytic and ATP-binding domain (HATPase-C) and a histidine kinase phospho-acceptor domain (HisKA); the receiver domain Sm0737 is composed of a receiver domain and an EAL domain (Figure 5a). According to the NCBI BLASTP analysis, no protein homologous to Sm0737 was found in *Xanthomonas* species, indicating that Sm0737 may play a specific role in *S. maltophilia*.

### 3.7. The Effect of Sm0737 and Sm1912 on Physiological Functions

The deletion of the *Sm0737* gene slightly reduced biofilm production compared to WT strains cultured at 15, 25, and 37 °C, respectively. No obvious changes were observed in the ∆*Sm1912* mutant compared to the WT strain when cultured at different temperatures (Figure 5b). Similar to its effect on biofilm formation, protease activity decreased slightly in the ∆*Sm0737* mutant strain at different temperatures, and almost no changes were detected in the ∆*Sm1912* mutant strain (Figure 5c).

To avoid the possibility that differences in growth rates may affect biofilm synthesis and protease activity, we generated growth curves for these strains. The strains started at the same cell density at an OD_600_ of 0.2, and no significant differences in growth rates were observed among these strains during the culture period (Figure 5d). 

### 3.8. Enzymatic Activity Was Not a Vital Contributor to c-di-GMP Levels

**Figure 6:**
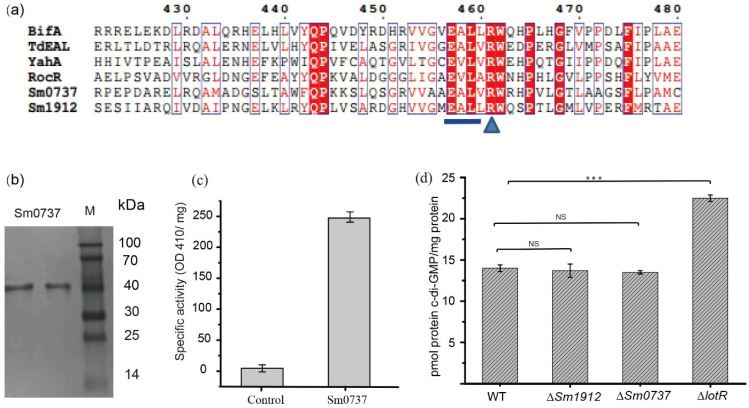
Detection of intracellular c-di-GMP levels and an enzymatic activity assay. (**a**) Sequence alignment of Sm0737 and Sm1912 with other proteins that have proven PDE activity. (**b**) SDS-PAGE analysis of the His6-tagged Sm0737 protein purified using nickel affinity chromatography. (**c**) Phosphodiesterase activity of the purified Sm0737 using colorimetric assays. (**d**) Intracellular c-di-GMP levels in the wild-types, Δ*Sm0737*, Δ*Sm1912*, and Δ*lotR* strains cultured at 25 °C for 48 h. Asterisks *** represent a statistically significant difference between control and experiments at *p* < 0.001, NS for non-significant difference between control and experiments.

The alignment of Sm0737 and Sm1912 with other known EAL-containing proteins (RocR (PDB ID: 3SY8) and BifA (WP-0030911007) from *Pseudomonas aeruginosa*, TdEAL (PDB ID: 2R6O) from *Thiobacillus denitrificans*, and YahA from *Escherichia coli.*) with PDE activity showed that the amino acid segments in the EAL motif and c-di-GMP binding site (aminio acid R) were highly conserved. This motif was intact in the Sm0737 and Sm1912 protein sequences, indicating that the Sm0737 and Sm1912 proteins were likely to be enzymatically active (Figure 6a).

SDS-PAGE revealed that purified Sm0737 produced a single band of the expected size of 43.8 kDa (Figure 6b). Incubation of purified Sm0737 with the model substrate bis(p-nitrophenyl) phosphate resulted in the production of the homology product p-nitrophenol, which could be detected at 410 nm; no product was detected in the control (Figure 6c). 

### 3.9. Detection of Intracellular c-di-GMP Levels in WT and Various Deletion Strains

Figure 6d shows the level of intracellular c-di-GMP among the various strains investigated in this study. The results showed that c-di-GMP levels were not obviously different between the WT, ∆*Sm0737*, and ∆*Sm1912* mutant strains, despite the fact that Sm0737 possesses c-di-GMP degradation activity, whereas LotR protein with no c-di-GMP degradation activity significantly increased c-di-GMP levels. This implies that a small c-di-GMP pool may not affect the overall c-di-GMP level in these strains. The enzymatic activity of c-di-GMP-related metabolism proteins may not be a vital contributor to changes in c-di-GMP levels, thus influencing physiological functions.

### 3.10. TCSs Regulated Biofilm Formation in Multiple Pathways

**Figure 7:**
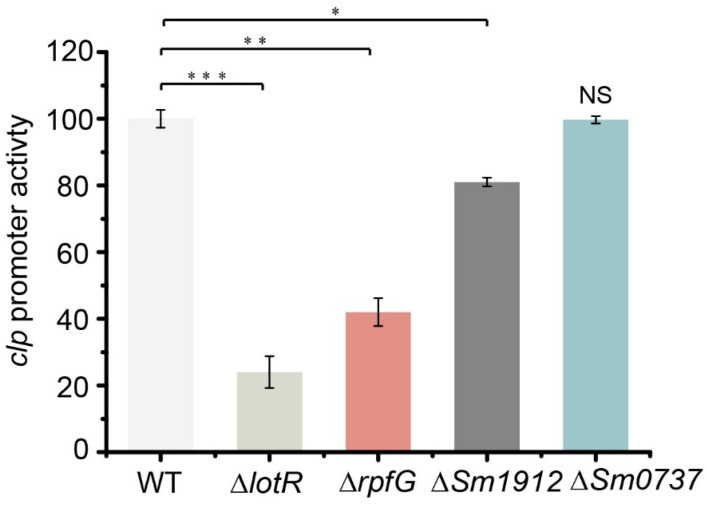
The promoter activity of *clp* in four TCS-regulator mutant strains. β-glucuronidase (gusA) activity was determined in the wild-type (WT), Δ*lotR*, Δ*rpfG*, Δ*Sm0737*, and Δ*Sm1912* strains. The strains carrying the fusion plasmid were cultured at 25 °C in LB for 48 h. Asterisks *, **, or *** represent a statistically significant difference between control and experi-ments at *p* < 0.05, *p* < 0.01, or *p* < 0.001, respectively.

To determine the effect of four c-di-GMP-related proteins on Clp expression, the promoter activity of *clp* was investigated in the respective mutants. The results revealed that β-glucuronidase activity was significantly reduced in Δ*lotR* mutants (78%), while a considerable reduction (58%) was observed in Δ*rpfG* mutants and a slight reduction in the *Sm0737* deleted strain. No obvious effect was caused by Sm1912/Sm1911 (Figure 7). These data combine the results of biofilm amounts in Δ*clp* and TCSs, indicating that LotS/LotR controlled biofilm formation in a Clp-mediated manner, whereas both RpfC/RpfG and Sm0712/Sm0711 acted in a distinct pathway.

## 4. Discussion

*S. maltophilia* survives at a broad range of temperatures as a ubiquitous bacterium, and its proteolytic and biofilm formation abilities are two critical determinants for evaluating its spoilage potential or pathogenicity. In this study, we found that temperature differentially controlled the protease production and biofilm formation of the *S. maltophilia* FF11 strain. When the temperature increased from 15 °C to 37 °C, a significant increase in biofilm production was observed (Figure 1a); in contrast, protease activity was reduced significantly within this range, especially from 25 °C to 37 °C (Figure 1c). *Stenotrophomonas* were the main microbial sources for cold-resistant bacteria resident in quick-frozen products [14,44]. The higher level of proteolytic enzymes of this organism at low temperatures may hydrolyze many proteins into peptides and free amino acids in foods. Hydrolysis products not only function as substrates for further growth of other microorganisms but also increase the level of biogenic amines due to the action of amino acid decarboxylases, thus contributing to food spoilage [45,46,47]. *Stenotrophomonas* biofilm formation occurs on food and clinical instruments and is difficult to remove once formed, which induces continuous contamination in food processing and clinical treatment. At present, the mechanism of biofilm formation and protease production in *Stenotrophomonas* is not well established.

Temperature is a universal environmental stimulus in food processing and clinical practice. As a typical example of an environment-origin opportunistic human pathogen, *S. maltophilia* thrives in broad temperature ranges from various environmental inhabits to the human body. In our study, we found that temperature differentially controlled protease production and biofilm formation in *S. maltophilia*, with increases in temperature inducing greater biofilm formation but reduced protease production (Figure 1). As an important, opportunistic human pathogen, *S. maltophilia* manifests resistance to most commercially available antimicrobials. Evidence showed that the susceptibility of the bacteria to antimicrobials is known to be affected by the temperature of incubation, probably as a result of changes in membrane fluidity and conformational changes of the bacterial outer membrane [48,49,50].

The cyclic AMP (cAMP) receptor protein Clp was proven to positively regulate low temperature-dependent protease synthesis in our previous study [36]. Here, we found that Clp negatively controlled biofilm formation, with a significant increase in biofilm biomass in *clp*-deleted strains. This evidence indicated that Clp differentially regulated protease production and biofilm formation (Figure 2). The secondary messenger cyclic di-guanosine monophosphate (c-di-GMP) is reportedly involved in food spoilage and biofilm formation by foodborne bacteria [51,52]. Bacteria can regulate a series of physiological processes via intracellular c-di-GMP, which perceives external stimuli [52]. In addition, it is generally accepted that TCS links environmental signals to cellular responses. The c-di-GMP metabolic proteins of the TCSs may contribute to the regulation of biofilm formation and protease production in response to temperature signals. Among these, both LotS/LotR and RpfC/RpfG in *S. maltophilia* played critical roles in positively mediating protease production. Nevertheless, these two TCSs exhibited a distinct effect on biofilm formation, a significant increase in biofilm biomass in *lotS* or *lotR* mutant strains, and a considerable reduction in *rpfC* or *rpfG* (Figure 3). Additionally, LotS/LotR contributed to various physiological functions, including EPS formation, bacterial swimming, and LPS synthesis, to a greater degree than TCS RpfC/RpfG (Figure 4). RpfC/RpfG as the critical TCS in quorum sensing devices could mediate the synthesis of various spoilage-related or pathogenic factors, including extracellular enzymes and EPS, biofilm formation, and bacterial motility, and has been well explored in many bacteria [53,54,55]. NCBI BLAST analysis revealed that both the *lotS* and *lotR* genes are widely distributed in the genomes of *Stenotrophomonas* spp., with amino acid similarities between 77 and 99%. These results prompted us to propose an alternative perspective: LotS/LotR, as a universal and global regulatory system, was involved in controlling various physiological functions related to food spoilage and clinical pathogenicity in *Stenotrophomonas*. These findings may provide clues to control the risk factors of *S. maltophilia* in food safety.

Among two other c-di-GMP-related TCSs, Sm1912 displays high sequence homology with RavR (83–85% identity), a regulator of *Xanthomonas*, a common plant pathogen, which is closely related to *S. maltophilia.* The TCS RavR/RavS may positively control extracellular enzyme production and EPS synthesis in *Xanthomonas* [43]. In *S. maltophilia*, we did not observe a significant change in protease production and biofilm synthesis in Sm1911- or Sm1912-deleted strains; some phenotypic differences were found between the mutant strains in these two genes, suggesting a distinct regulatory pathway of Sm1911/Sm1912 in different bacteria.

No homologous sequences of Sm0738/Sm0737 were found in the NCBI genomes of *Xanthomonas* species, implying that it may have a specific function in *S. maltophilia*. Sm0737 is a putative response regulator containing the REC and EAL domains. Sequence alignment of several characterized proteins, including EAL domains with confirmed PDE activity, showed that the predicted EAL domain of Sm0737 was highly conserved (Figure 6a). Enzyme activity analysis revealed that Sm0737 displayed strong PDE activity (Figure 6c). It has been shown that the conserved residues “E” and “L” in the EAL domain are extremely important for the PDE activity of c-di-GMP [56]; however, the residue “A” in the motif can be functionally replaced by other hydrophobic amino acid residues [34].

Although both RpfG (well-defined PDE activity) and Sm0737 were confirmed to have PDE activity that can degrade c-di-GMP into GMP, some phenotypic differences were found between these mutant strains in the two genes. We speculate that the enzymatic ability to degrade c-di-GMP may differ between strains. Another reason for any difference may be because proteins can perform their regulatory functions via physical interactions with other proteins, independent of their c-di-GMP PDE activity. Similarly, the regulator protein LotR, containing a GGDEF or EAL domain, lacks apparent DGC or PDE activity yet still affects the c-di-GMP level (Figure 6d), suggesting a possible direct interaction with other proteins involved in c-di-GMP metabolism. These results indicated that the enzymatic activity of c-di-GMP-related proteins may not be a vital contributor to changes in c-di-GMP levels, further influencing physiological functions. 

A significant increase in biofilm level was observed in both TCS LotS/LotR (3.5–3.9-fold) and Clp (4.1-fold) deleted strains, while the transcription level of *clp* appeared to be reduced by ~78% in the *lotR* mutant strain. These results indicate that LotS/LotR controlled biofilm formation in a Clp-mediated manner, with a similar effect on protease production. Both RpfC/RpfG and Sm0712/Sm0711 negatively regulated biofilm formation, in contrast to the protease production trend, indicating that these two TCSs control biofilm formation via a distinct pathway, although we observed a reduction of *clp* transcription level in two TCSs regulator mutant strains (Figure 7). This research reveals regulation by c-di-GMP-related TCSs and transcription factor Clp in controlling pathogenic or spoilage factors of *S. maltophilia* through environmental temperature and improves our understanding of c-di-GMP-related proteins in mediating cell processes.

## 5. Conclusions

Protease activity and biofilm-forming ability were vital virulence factors and spoilage determinants in *S. maltophilia.* In this study, we determined the regulatory factors or potential mechanisms for biofilm formation and protease production in this species. Firstly, we found that temperature differentially regulated biofilm formation and protease production and that Clp could negatively regulate thermosensor biofilm formation, in contrast to protease production. Secondly, the transcription level of *clp* was determined in four c-di-GMP-related TCSs, and the results indicated that *clp* levels were predominantly controlled (~78%) by LotS/LotR, partially controlled by both RpfC/RpfG (~58%) and a novel TCS Sm0738/Sm0737 (~18%), with no obvious effect caused by Sm1912/Sm1911. The ability of biofilm formation in Δ*clp* and ΔTCSs strains suggested that LotS/LotR controlled biofilm formation, mostly in a Clp-mediated manner, whereas both RpfC/RpfG and Sm0738/Sm0737 may function in a distinct pathway. Moreover, evidence indicates that the enzymatic activity of c-di-GMP-related metabolism proteins may not be a vital contributor to the changes in c-di-GMP levels overall, thus influencing various cellular functions. Our results elucidate the correlation between regulation of c-di-GMP-related TCSs and Clp in controlling cellular processes involved in food spoilage and pathogenicity in *Stenotrophomonas* and provide clues to control related risk factors of *S. maltophilia* in food safety.

## Figures and Tables

**Table 1:** Strains and plasmids used in this study.

Strain or Plasmid	Description	Reference or Source
**Strains**		
*Stenotrophomonas moltophilia* FF11	Wild-type(FF11)	Lab collection
∆*lotR* or ∆*lotS* mutant	*lotR* or *lotS* gene deletion mutant derived from FF11, Cmr	Previous study
∆*rpfG* or ∆*rpfC* mutant	*rpfG* or *rpfC* gene deletion mutant derived from FF11, Cmr	Previous study
∆Sm1912 mutant	*Sm1912* gene deletion mutant derived from FF11, Cmr	This study
∆Sm0737 mutant	*Sm0737* gene deletion mutant derived from FF11, Cmr	This study
∆*lotR* (C) or ∆*lotS* (C)	Complement of *lotR* in ∆*lotR* mutant, Te	This study
∆*rpfG* (C) or ∆*rpfC* (C)	Complement of *rpfG* in ∆*rpfG* mutant, Te	This study
∆Sm1912 (C)	Complement of Sm1912 in ∆Sm1912 mutant, Te	This study
∆Sm0737 (C)	Complement of Sm0737 in ∆Sm0737 mutant, Te	This study
*E. coli*		
JM109	RecA1, endA1, gyrA96, thi, supE44, relA1, Δ(lac-proAB)/F′ traD36, lacI q, lacZ	Novagen
S17-1 λ pir	λ pir lysogen of S17-1 [thi pro hsdR2 hsdM+ recA RP4 2-Tc::Mu-Km::Tn7 (Tp^r^ Sm^r^)]; permissive host able to transfer suicide plasmids requiring the pir protein by conjugation to recipient cells	Novagen
**Plasmids**		
pEX18Tc	Low copy number plasmid; For gene knockout; Tc	HonorGene
pLAFR3	Broad host range cloning vector, RK2 replicon, Mob+, Tc^r^	Bio Sci

**Table 2:** Primers used in this study.

Primers	Sequence (5′ to 3′)
1912 up F	CGC***GGATCC***CTGGAAACCAGCAACCGCGAG
1912 up R	GGGGTGGTGCTGCCGCGACCACGCAGGTAGGAC
1912 cmr F	GGTCGCGGCAGCACCACCCCGTCAGTAGCTGAA
1912 cmr R	ATGTGCTTGCTTACGCCCCGCCCTGCCACTCAT
1912 down F	CGGGGCGTAAGCAAGCACATCCACCAGGCGTTC
1912 down R	CCC***AAGCTT***TGCCGTCGGAGTTGAACCAGGTC
0737 up F	CCG***GAATTC***ATGACCCAGCGTGTCCTGATCCT
0737 up R	GGGGTGGTGCCGCAACAGCTCGCCTTCCAG
0737 cmr F	AGCTGTTGCGGCACCACCCCGTCAGTAGCTGAA
0737 cmr R	GCGTTTCATCTTACGCCCCGCCCTGCCACTCAT
0737 down F	CGGGGCGTAACTTCCGGGTGCCGGTTTCCATC
0737 down R	CGC***GGATCC***ACGGCCCGTGGCGTTTCATC
pLAFR3-0737 F	CATG***CCATGG***TGACCCAGCGTGTCCTGATC
pLAFR3-0737 R	CCG***CTCGAG***TCAACGCAGGACGGCCCGTG
pL6rmlA-gusA F	CCG***GAATTC***TACTGGGTGGTCTTGAAGGTGTCG
pL6rmlA-gusA R	CGG***GGTACC***TGTGGGCACTACCTGTTCTCCTG

Enzyme digestion sites were labeled with ***blackbody***.

## Data Availability

The original contributions presented in the study are included in the article, further inquiries can be directed to the corresponding author.

## Abbreviations

DGC—diguanylate cyclase; EPS—exopolysaccharide; HisKA—histidine kinase phospho-acceptor; HPLC—high-performance liquid chromatography; LPS—lipopolysaccharide; ORF—open reading frame; TCS—two-component system; WT—wild-type.
